# The Discovery of Novel Experimental Therapies for Inflammatory Arthritis

**DOI:** 10.1155/2009/698769

**Published:** 2010-02-18

**Authors:** Charles J. Malemud

**Affiliations:** Division of Rheumatic Diseases, Department of Medicine, University Hospitals Case Medical Center, Case Western Reserve University School of Medicine, Cleveland, OH 44106, USA

## Abstract

Conventional and biologic disease-modifying antirheumatic drugs have revolutionized the medical therapy of inflammatory arthritis. However, it remains unclear as to what can be done to treat immune-mediated chronic inflammation after patients become refractory to these therapies or develop serious side-effects and/or infections forcing drug withdrawal. Because of these concerns it is imperative that novel targets be continuously identified and experimental strategies designed to test potential arthritis interventions in vitro, but more importantly, in well-validated animal models of inflammatory arthritis. Over the past few years, sphingosine-1-phosphate, interleukin-7 receptor, spleen tyrosine kinase, extracellular signal-regulated kinase, mitogen-activated protein kinase 5/p38 kinase regulated/activated protein kinase, micro-RNAs, tumor necrosis factor-related apoptosis inducing ligand and the polyubiquitin-proteasome pathway were identified as promising novel targets for potential antiarthritis drug development. Indeed several experimental compounds alter the biological activity of these targets and have shown clinical efficacy in animal models of arthritis. A few of them have even entered the first phase of human clinical trials.

## 1. Introduction

 The therapy of various types of inflammatory arthritis, including, adult rheumatoid arthritis (RA), juvenile idiopathic arthritis (JIA), psoriatic arthritis and ankylosing spondyloarthopathy that were previously treated with glucocorticoids, nonsteroidal antiinflammatory drugs, gold and bed rest was revolutionized by the introduction of the conventional disease-modifying antirheumatic drugs (DMARDs), hydroxychloroquine, sulfasalazine, leflunomide, azathioprine, cyclosporine, minocycline and methotrexate monotherapy or several combined conventional and biologic DMARD therapies into the standard of medical care [1–3]. The advent of the use of biologic DMARDs, including the 5 forms of anti-TNF-*α*-receptor blocking molecules, infliximab, adalimumab, certolizumab pegol, golimumab and etanercept [4–7], interleukin-1-receptor antagonist (IRAP) [8, 9], results from recent clinical trials with canakinumab, a fully human monoclonal antibody that neutralizes the bioactivity of human IL-1*β* [10], abatacept, a selective modulator and inhibitor of the T-cell costimulatory molecule CTLA-4 [11–13] and the monoclonal antibody, rituximab which deletes a subset of immature and/or memory B-cells expressing the CD20 cell surface protein marker [14–16] has added to the armamentarium of useful drugs to treat patients with inflammatory arthritis. Additionally, the positive results obtained from recent clinical trials in adult RA and JIA patients indicated that additional novel agents such as the anti-IL-6 receptor (IL-6R) blocking monoclonal antibody, tocilizumab [17, 18], which has been approved for use in Europe, India and Japan are likely to be approved in the United States by the Federal Drug Administration in the not too distant future. Nevertheless, there is compelling evidence which indicates that patients with inflammatory forms of arthritis can become unresponsive or refractory to the currently available conventional DMARDs and/or biologic DMARDs and develop serious side-effects including neutropenia, auto-antibodies, antiidiotypic antibodies (even to some anti-TNF-*α* or anti-IL-6R monoclonal antibodies characterized as fully humanized) as well as reactivation and/or the onset of new bacterial infections especially at higher than recommended anti-TNF-*α* doses which may require withdrawing the drug [4, 19–24]. These findings in the biologic DMARD postmarketing surveillance period likely necessitates that new antirheumatic targets be continuously identified. Experimental agents that interact with these newly discovered targets must be thoroughly examined for their effects in vitro as well as in animal models of inflammatory arthritis prior to any consideration of their use in assessments of safety, efficacy and disease-modifying activity in patients with inflammatory arthritis.

 A previously published compilation of review articles highlighted the recent advances in the identification of novel targets and cellular processes in RA, which included non-IL-1, non-TNF-*α* and non-IL-6 proinflammatory cytokines, chemokines, adhesion molecules, vascular endothelial growth factor and insulin-like growth factor-1, matrix metalloproteinases (MMPs), complement and pro- and antiapoptosis molecules [25] as well as the Janus kinase/Signal Transducers and Activators of Transcription (JAK/STAT) pathway whereby the Jak3 inhibitor, CP690550 is the first small molecule inhibitor (SMI) to reach phase III clinical trials status [26], Mitogen-Activated Protein Kinase (MAPK) pathway [27, 28], and the IL-6/IL-6 receptor/gp130 complex [29, 30], all of which are applicable to additional basic and clinical studies wherein their future use in the medical intervention of adult and childhood RA and other inflammatory arthopathies may be further assessed. 

 This review will critically evaluate the extent to which several molecules, including, sphingosine-1-phosphate (S1P), IL-7-receptor (IL-7R), spleen tyrosine kinase (SyK), MEK/ERK 1/2, Mitogen-activated protein kinase 5/p38 kinase regulated/activated protein kinase (MK5/PRAK), microRNA (miRNA), tumor necrosis factor-related apoptosis-inducing ligand (TRAIL) and inhibitors of proteasome activity, appear to be promising novel experimental targets for future drug development for the treatment of inflammatory arthritis.

## 2. Sphingosine-1-Phosphate (S1P)

Sphingosine-1-phosphate (S1P) is a signaling sphingolipid and a bioactive lipid mediator [31]. Although once only recognized as regulator of angiogenesis, vascular homeostasis and permeability [31], the most recent evidence indicated that S1P was a critical regulator of T-cell and B-cell trafficking [32] and macrophage function [33]. Thus, the binding of S1P to its receptors, S1PR1/S1PR2 [34], triggers and is required for stimulating the movement of immune cells from the thymus and lymph nodes into lymphatic vessels from where they can travel through peripheral circulation to synovial joints. Additionally, it was shown that the secreted form of S1P also regulated cell survival and apoptosis by its capacity to bind to and activate 5 specific G protein-coupled receptors, S1P1-S1P15 [35]. 

 S1P is produced by the activation (i.e., phosphorylation) of sphingosine (Sph) via its 2 isoforms of sphingosine kinase-1/2 (SphK-1/2) resulting in the production of the prosurvival S1P molecule with decreasing levels of the proapoptotic Sph molecule [35]. Thus, inhibiting the formation of S1P either by targeting SphK, or by inhibiting the binding of S1P to S1PR1/S1PR2 is likely to result in a reduced egress of activated T-cell and B-cell from lymphoid tissues and presumably fewer activated lymphocytes making their way into the synovium. These strategies may even be useful for also targeting apoptosis resistance which is a common hallmark of RA synovium [36, 37].

 What is the current evidence that altering the activity of S1P would provide significant and beneficial effects by restricting some of the pathophysiologic events that contribute to inflammatory arthritis? Firstly, aberrant synovial cell proliferation is a signature event in RA [38]. Thus, it is likely that studying cell lines with an abnormal capacity for proliferation might be useful in dissecting out the role of S1P in regulating DNA synthesis. In that regard, several murine F9 embryonic carcinoma cell lines were produced with varying expressional levels of the S1P-degrading enzyme, S1P lyase (SPL) and/or SphK1 [39]. F9 cells overexpressing SphK1 exhibited elevated DNA synthesis, whereas other S1P-accumulating cells or SPL-null cells overexpressing SphK1 did not. These studies suggested that it was SPL, rather than S1P, that regulated mitogenesis in this cell line. Furthermore, genetic studies in mice expressing reduced levels of SPL showed decreased circulating lymphocytes as a result of alterations in lymphocyte trafficking and studies of lymphoid tissues following oral administration of 2-acetyl-4(5)-(1(R), 2(S), 3(R), 4-tetrahydroxybutyl)-imidazole, an experimental inhibitor of SPL, showed a clear relationship between reduced SPL activity, elevated S1P levels, and lower levels of circulating lymphocytes [40], thus supporting the targeting of S1P signaling as a potentially important antiinflammatory pathway [41]. 

 Of note, on August 27, 2009, Lexicon Pharmaceuticals announced that an orally-administered SMI of SPL, 2-acetyl-4(5)-(1(R), 2(S), 3(R), 4-tetrahydroxybutyl)-imidazole (i.e., LX2931), was being assessed in a Phase 2 multicentered randomized, double-blinded placebo-controlled clinical trial in the US and Eastern Europe on subjects with active RA who were also receiving methotrexate [42]. The ACR20 response to LX2931 will be the primary study endpoint. In the Phase 1a clinical trial conducted on normal volunteers, a dose-dependent decrease in absolute lymphocyte count and a maximal effect correlating with the plateau in systemic exposure at doses of 100 to 125 mg of LX2931 was observed. An episode of acute abdominal pain which resolved within 24 hours was observed in 2 of 24 subjects in this single ascending-dose trial when subjects received doses of LX2931 above 175 mg, a dose which potentially represented (according to the manufacturer) a dose-limiting tolerability level.

 Another line of evidence suggesting a role for S1P in RA stems from studies in which TNF-*α* induction of COX-2 activity and PGE_2_ synthesis was studied in L929 fibroblasts [43]. Of note, S1P induced COX-2 and PGE_2_ in a dose-dependent (100–300 nM) manner. Small interfering RNAs (siRNA) directed against SphK decreased SphK1 protein and inhibited TNF-*α*-induced SphK activity. Interestingly, siRNA directed against S1P or S1P phosphatase (SPP) also enhanced COX-2 and PGE_2_ production, whereas siRNA directed against SphK1 inhibited the effects of exogenous Sph and ceramide on the induction of PGE_2_ indicating that it was the intracellular metabolism of S1P that regulated COX-2 and PGE_2_ induction. However, the extent to which experimental strategies of this kind will produce similar results when employed in cultures of normal synovial fibroblasts or RA fibroblast-like synoviocytes (RA-FLS) has not been established. It is also noteworthy that silencing of SPP2 by siRNA also led to a marked reduction in TNF-*α*-induced IL-1*β* and a partial reduction in IL-8 in endothelial cells [44] suggesting that the targeting of SPP2 may also effectively limit the proinflammatory role of S1P.

## 3. IL-7 Receptor

**Figure 1:**
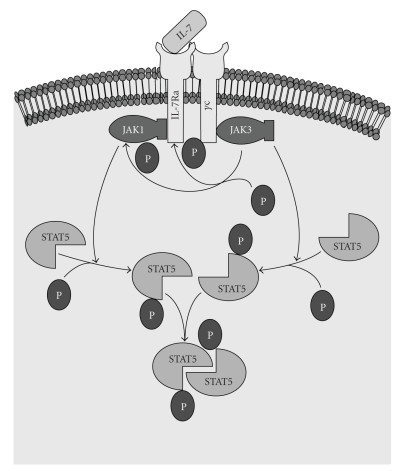
The IL-7/IL-7R Pathway. The IL-7R is composed of a *γ*C and R*α* polypeptide. The R*α* component is unique to the IL-7R, whereas *γ*C is common to several cytokine-mediated pathways, including IL-2, IL-4, IL-9, IL-15 and IL-21. The binding of IL-7 to R*α* results in the dimerization of *γ*C and R*α*. JAK3 is associated with *γ*C. After dimerization of *γ*C with R*α*, JAK3 can phosphorylate R*α* and/or JAK1. Activation of JAK3 results in phosphorylation of STAT5. Phosphorylation of STAT5 is required for STAT5 to act as a transcription factor (reviewed in [26]).

 Expression of the IL-7 receptor (IL-7R) gene (also known as CD127) plays a central role in thymocyte development [45], T-cell survival, B-cell maturation, T-cell-dendritic cell (DC) interactions [46, 47], as an inducer of lymphoid tissue development [48] as well as being useful for identifying T-regulatory (*T*_reg_) cells producing the FoxP3 phenotype [49]. To function normally, the IL-7R requires the presence of the IL-2 receptor gamma chain (IL-2R*γ*) which is the common *γ*-chain (Figure 1) that is shared by the receptors of various cytokines including IL-2, -4, -7-,-9 and -15, -21. In that context, IL-2R*γ* has been reported to be critical for V(D)J recombination during lymphocyte development [50]. IL-7R was also found to important in regulating the accessibility of the T-cell receptor (TCR) *γ*-locus (TCR-*γ*) by STAT5 and histone acetylase [51]. Thus, overexpression of IL-7R*α* is likely to be highly relevant to the pathogenesis and even to the progression of inflammatory arthritis. 

 For example, it was shown that murine T-cell and B-cell development was dependent on IL-7R*α*, in that forced expression of the antiapoptosis gene Bcl-2 in murine T-cells from IL-7R*α* deficient mice resulted in restoration of thymic positive selection and normal T-cell numbers and function [52]. Interestingly, a deficiency in the proapoptosis gene, Bax, also partially corrected IL-7R*α* deficiency and the BH3-domain containing apoptosis proteins Bad and Bim were implicated as contributing to the apoptosis pathway suppressed by IL-7/IL-7R [53]. Additionally, in contrast to IL-7R null mice, Flt3 null mice crossed with IL-7R null mice failed to produce mature B-cells or for that matter, B-cell precursors during fetal development [54]. Taken together, these results indicated that IL-7R*α* was required for normal T-cell and B-cell development. Thus, defective or overexpressed IL-7/IL-7R*α* signaling could be critical in the regulation of other receptor-mediated events that regulate apoptosis as well as immune-mediated events that contribute to inflammatory arthritis.

 Several criteria needed to be met in order for IL-7R to be further considered as a promising target for experimental intervention in inflammatory arthritis. First and foremost would be evidence for elevated levels of IL-7 in the synovial fluid of joints with inflammatory arthritis. A recent review of the literature by Churchman and Ponchel [55] indicated that this indeed was the case. However, one of the first indications that IL-7 was likely to play a critical role in experimental inflammatory arthritis also stemmed from the earlier studies by Sawa et al. [56] who showed that mice homozygous for the F759 mutation in the gp130/IL-6R subunit showed elevated Stat3 activation and a rheumatoid-like arthritic joint disease. Furthermore, the gp130 mutation resulted in elevated levels of IL-7. In turn, conditional knockout of Stat3 in nonlymphoid cells showed that the increased levels of IL-7 in these mice were Stat3-dependent. Anti-IL-7 antibody not only inhibited CD4^+^ T-cell proliferation (which was a requirement for the development of arthritis) but anti-IL-7 antibody administration also ameliorated arthritis severity. Thus, arthritis produced in these mice by the F759 mutation in gp130 required not only homeostatic CD4^+^ T-cell expansion but upregulation of IL-7 gene expression in nonhemopoietic cells as well.

 Turning to studies in human RA, Kim et al. [57] showed that elevated levels of IL-1*β* and TNF-*α* in RA synovial fluid typically increased stromal cell production of IL-7 in vitro. In turn, IL-7 was also shown to upregulate production of TNF-*α* in monocytes. Of note, IL-7 was shown to be an important inducer of osteoclastogenic cytokines in T-cells by RANKL, the latter a strong promoter of bone destruction in RA. Most importantly, this event was apparently independent of TNF-*α* [58], suggesting that anti-TNF-*α* therapies would be unlikely to completely abrogate IL-7-mediated cellular events in RA. However, van Roon et al. [59] showed in vivo that TNF-*α*R blockade did block IL-7 production in those RA patients that responded to anti-TNF-*α* therapy, but high IL-7 levels persisted in anti-TNF-*α* nonresponders. More recently, Hartgring et al. [60] also found significantly higher levels of IL7R*α* in the synovial fluid from RA and undifferentiated arthritis patients which strongly correlated with elevated levels of IL-7 and increased numbers of the CD3^+^ T-cell subset. Interestingly, a large number of B-cells and macrophages also expressed IL-7R*α* although this was less significant than was found for T-cells. Additionally, there was a strong correlation between IL-7R*α*-expressing T-cells that also failed to express FoxP3 which showed marked proliferation in vitro compared to IL-7R*α*-expressing T-cells that expressed FoxP3. Experimental e*x vivo* studies on monocytes collected from RA patients when treated with recombinant human soluble IL-7R*α* in vitro inhibited IL-7-induced T-cell proliferation and interferon-*γ* (IFN-*γ*) production, suggesting that IL-7R*α* blockade might of potential importance as an alternative strategy to TNF-*α* blockade for limiting IL-7-induced RA immunopathology. This point was also stressed by Vudattu et al. [61] for treating patients with autoimmune diseases associated with central nervous system inflammation.

## 4. Spleen Tyrosine Kinase

**Figure 2:**
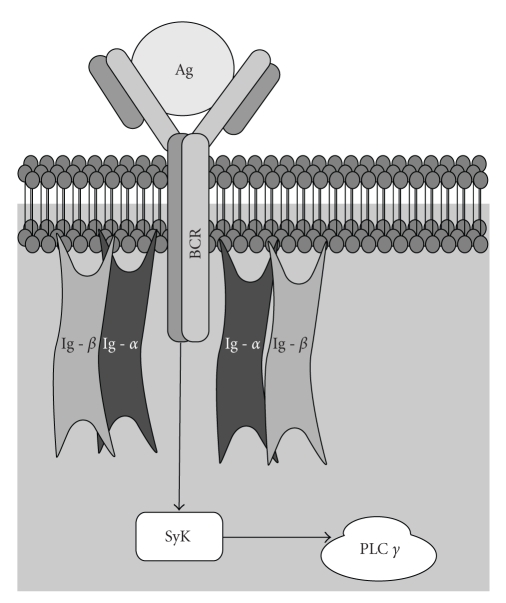
The Role of Syk in Antigen-Mediated Activation of the B-cell Receptor. The B-cell receptor (BCR) is composed of a ligand binding moiety that interacts with antigen (Ag) and a signaling moiety in the form of the heterodimer, Ig-*α*/Ig-*β* complex, (also known as CD79) which is held together by disulfide bridges. Each component of the Ig-*α*/Ig-*β* heterodimer complex spans the plasma membrane and also possesses a cytoplasmic tail component containing the immunoreceptor tyrosine-based activation motif (ITAM). In this example, the binding of Ag to BCR triggers phosphorylation of Syk. Activation of Syk regulates phospholipase C*γ* (PLC*γ*) activity. PLC*γ*2 was recently shown to be required for the formation, maintenance and survival of memory B cells within germinal centers [67].

 Spleen tyrosine kinase (SyK) (Figure 2) and *ζ*-chain associated protein-70 (ZAP-70) are nonreceptor kinases that are primarily expressed in hemopoietic cells, including cells of the spleen, mast cells, neutrophils and macrophages [62]. Syk and ZAP-70 are also involved in T-cell and B-cell receptor signaling potentially making Syk and ZAP-70 enzyme targets for the treatment of autoimmune diseases [63]. Of note, since Cbl ubiquitin ligase was previously shown to be a negative regulator of Syk [64], various experimental attempts have been made to limit SyK activation through targeting of the polyubquitination-proteasome pathway [65, 66].

 Because of its apparent critical role in regulating T-cell and B-cell expansion and the proliferation of cells containing the F*γ*-activating receptor as well as mediating immunoreceptor signaling in inflammatory cells and immune complex-mediated signal transduction, SyK must be considered a promising target for designing interventional drugs for the treatment of immune-mediated inflammatory arthritis. At the experimental level, Pine et al. [68] showed that R788 a prodrug of the active novel SyK small molecule inhibitor R406 suppressed the severity of arthritis, bone erosions, pannus development and synovitis in murine collagen-induced arthritis (CIA). The reduced expression of SyK in the R788-treated mice correlated with an amelioration of clinical arthritis, a reduction in proinflammatory chemokines and cytokines, including the CXCR2 ligand KC-GRO-*α*, macrophage chemoattractant protein-1 (MCP-1), IL-1, and IL-6, as well as inducing suppression of cartilage oligomeric matrix protein release, the latter protein a sensitive in vitro biomarker for articular cartilage extracellular matrix degradation. 

 Because of the apparent successful preclinical response to R788 in the well-validated CIA animal model of RA, a Phase II clinical trial involving, fostamatinib (R406), was conducted. Fostamatinib effectively improved clinical RA response rates within a 3 month treatment period with gastrointestinal side-effects, principally diarrhea, and neutropenia, which was related to the fostamatinib dose employed, the most commonly reported adverse events [69]. In addition to improving clinical outcomes measurements as determined by the American College of Rheumatology (ACR) criteria as early as 1 week after oral administration, R406 also reduced the level of serum IL-6 and MMP-3 (stromelysin-1) in the groups receiving 100 mg and 150 mg of R406 twice daily [70]. However, it remains to be determined as to the extent that R406 can be continuously employed in the treatment of chronic RA, the extent to which reduced levels of SyK are maintained by administration of R406 and whether R406 therapy will show a sustained improvement in arthritis symptoms compared to that obtained with conventional or biologic DMARDs.

## 5. MEK/ERK 1/2

 ERK 1/2 belongs to the SAP/MAPK family of protein kinases. ERK 1/2 must be activated before it can act as a fully activated protein kinase and as such the activation of an MAPK such as ERK 1/2 is generally carried out by one of at least 7 upstream MKK proteins [71]. Moreover, MKK activity is also regulated by further upstream MKKK and MKKKK activity that are either tyrosine or serine-binding proteins which may also require low molecular weight GTP-binding proteins for MKKK activation. Much of the accumulated evidence has indicated that the MKK-MAPK activated protein kinase complex is further organized by additional scaffolding proteins that provide the structural requirements for specific MKKK activation selectivity for the action of GTPases, additional protein kinases and receptors [72]. 

**Figure 3:**
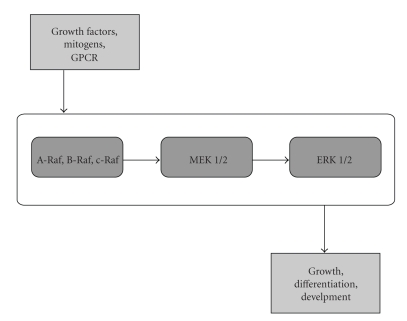
Activation of MEK 1/2 and ERK 1/2 by MAP3K. The phosphorylation (i.e., activation) of A-Raf, B-Raf or c-RAF, by MAPKKK (i.e., MAP3K) results in MAP2K-mediated phosphorylation of MEK 1/2 and downstream activation of ERK 1/2 (reviewed in [27]).

 MEK is the key regulatory protein kinase activation in the Ras/Raf/MEK/ERK pathway (Figure 3). In that regard, MEK is critical for the upregulation of several proinflammatory cytokines, including, TNF-*α*, IL-1*β* and IL-6 [73]. Additionally, in the context of inflammatory arthritis, recent studies using the novel MEK SMI AZD6244 showed that AZD6244 inhibited osteoclastic differentiation, function and cytokine production in dentine disc cultures [74] whereas a previous study showed that 2 MEK SMIs, U0126 and PD98059, blocked osteoclast development from the RAW264.7 preosteoclastic cell line [75]. These results suggested that MEK blockade could be useful for attenuating proinflammatory cytokine synthesis as well as for suppressing osteoclast-mediated bone erosions in inflammatory arthritis. However, the “cross-talk” that is known to occur between the JAK/STAT and MEK/ERK pathway [28, 76] likely also creates an added layer of complexity for regulating osteoclast function in inflammatory arthritis since the JAK/STAT pathway has also been considered as a suitable target pathway for the suppression of inflammatory disorders [26]. In that regard, Kwak et al. [77] showed that AG-490, an inhibitor of Jak2/Jak3 activity, actually induced osteoclast survival by activating both the MEK/ERK and PI3K/PTEN/Akt cell survival pathway. Thus, it is worthwhile considering the possibility that inhibition of osteoclast function using other novel strategies that target RANKL [78, 79] may also be required in this context.

 Compelling evidence arising from malignant transformation studies in which MEK SMIs were employed in vitro has also implicated Raf/MEK/ERK [80] as a component of a “cross-talking” network with the PI3K/PTEN/Akt cell survival pathway [81]. The results of these studies suggested, however, that inhibition of MEK/ERK may have only limited therapeutic benefit for dampening synoviocyte proliferation and hyperplasia that is a hallmark of rheumatoid synovial pannus. Nevertheless, Thiel et al. [82] showed that the selective MEK SMI, PD184352 suppressed murine CIA paw edema and clinical arthritis scores while also reducing IL-1*α*-induced activated ERK levels in human synovial fibroblasts as well as inhibiting proteoglycan loss from articular cartilage slices in vitro. Another experimental ERK inhibitor, FR180204, also suppressed murine CIA arthritis in which amelioration of clinical arthritis by FR180204 may also involve reduced antigen-specific activation of murine T-cells [83]. However, in the first clinical trial of its kind in RA patients to employ a specific MEK SMI, ARRY-162, used in combination with a stable dose of methotrexate, none of the ARRY-162 treatment groups demonstrated a significant ACR20 response compared to placebo after 12 wks [84]. This result confirmed a previous European clinical trial with ARRY-162 which also failed to achieve a statistically significant outcome in clinical improvement. Thus, it remains to be determined if the “positive” results obtained in experimental inflammatory arthritis employing specific MEK/ERK SMIs can ever be translated into future use for the therapy of human RA.

## 6. Mitogen-Activated Protein Kinase 5/p38 Kinase Regulated/Activated Protein Kinase

 Mitogen-activated protein kinase 5 (MK5) also known as p38 kinase regulated/activated protein kinase (PRAK) is a 471 amino acid protein with a 20%-30% sequence identity to the cyclic AMP responsive element binding protein, CREB-phosphorylating MAPK-regulated protein kinase RSK-1, -2, -3 [88–90]. MK5/PRAK was found to be expressed in most human tissues and activated by cell-stressors and proinflammatory cytokines in vitro. In turn, PRAK activity was regulated by p38*α* and p38*β* activity. Once activated, MK5/PRAK was reported to directly phosphorylate heat shock protein 27 (Hsp27) [91, 92], the latter having been implicated in several physiologically relevant immune-mediated inflammatory responses such as CD8^+^ lymphocyte subset expansion and apoptosis resistance [93] as well as in the activation of the Toll-like receptor-4 in monocyte-derived RA DCs [94]. Additionally, the conventional DMARD, methotrexate, was shown to enhance PGD_2_-stimulated Hsp27-induction in MC3T3-E1 osteoblasts at a point downstream of MAPKs [93]. MK5/PRAK was also shown to activate atypical ERK 3/4 [95], but the significance of this pathway in inflammatory arthritis remains unclear. However, the MK5/PRAK promoters of human, mouse and rat all contain a cyclic AMP (cAMP) responsive element that binds to CREB in vitro [96], indicating that molecules involved in inflammatory arthritis that also activate cAMP-dependent protein kinase A (e.g., PGE_2_), may also upregulate MK5/PRAK transcription. 

 It was suggested that novel upstream p38*α*-specific SMIs, such as SD-282 (indole-5-carboxamide) could potentially be useful inhibitors of p38*α*-driven inflammation [97]. Thus, p38*α* inhibition by SD-282 may also affect downstream MK5/PRAK-driven p38 kinase-regulated pathways as well [98]. Additionally, Sun et al. [99] reported that PRAK activated the tumor suppressor p53 by direct phosphorylation. Although it has not been directly determined, PRAK may therefore also be also involved in p53-mediated transactivation activity of inflammatory response genes, such as PGE_2_ [100], as well as playing a role in the regulation of murine CIA responses [101]. In the latter, Type II collagen-stimulated T-cell activation and IFN-*γ* production ex vivo was significantly higher in p53 null mice with CIA compared to their wild-type counterparts [101]. An experimental inhibitor of MK5/PRAK, GLPG0259, was reported to provide “excellent” protection against bone erosion as well as reducing inflammation in an RA animal model [102].

## 7. Micro RNAs

**Figure 4:**
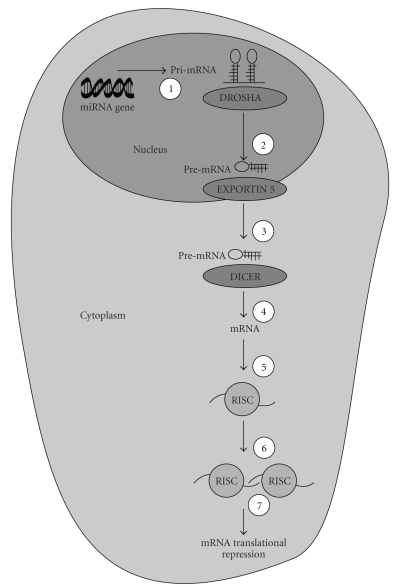
The miR Pathway. Step 1 of the miR pathway involves the transcription of a 70–100 nucleotide (nt) pri-miR from an miR gene which is then processed in the nucleus via Step 2 by the RNAse III enzyme, DROSHA to yield pre-miR. Following the transport of pre-miR from the nucleus to the cytoplasm mediated by the nuclear export receptor protein, EXPORTIN 5 at Step 3, a second RNAse III enzyme, DICER, digests pre-miR at Step 4 resulting in a 21–25 nt miR. At this juncture, miR has the capacity to bind to the RNA-Induced Silencing Complex (RISC) and at Steps 5 and 6 aligns itself with an mRNA. The final step (Step 7) is dependent on whatever complimentarity exists between a miR and a target sequence in mRNA. Therefore, the degree of complimentarity between a miR and an mRNA results in either translational repression or mRNA cleavage (reviewed in [109]).

Micro- RNAs (miRs) are small noncoding RNA molecules composed of double-stranded RNAs of 21–25 nucleotides derived from endogenously expressed transcripts with characteristic hairpin structures (Figure 4). miRs are known to negatively regulate gene expression at the posttranscriptional level [103, 104]. Lewis et al. [105] first proposed that perhaps as much as one-third of all mRNAs were targets for miR-mediated regulation. In the context of the putative epigenetic role for miRs in regulating the inflammatory response in RA [106], Taganov et al. [107] showed that miR-146a/b, miR-132 and miR-155 were endotoxin-responsive. Moreover, miR-146a was also found to be an NF-*κ*B-responsive gene with miR146a/b predicted to interact with sequences of the 3′ untranslated region of TNF-associated factor-6 (TRAF6) and IL-1 receptor-associated kinase-1 (IRAK-1) genes which are 2 key adaptor molecules intimately involved in the regulation of the Toll-like receptor (TLR) and cytokine receptor pathways. Thus, expression of the miR146a/b could be expected to alter the functional regulation of these signaling pathways. Another piece of the miR story with specific relevance to inflammatory arthritis emerged when O'Connell et al. [108] demonstrated that miR-155 was the only miR to be upregulated in monocyte cultures by either polyriboinosinic acid:polyribocytidylic acid [poly (I:C)], a synthetic ligand that activates the TLR pathway, and interferon-*β* (IFN-*β*). TLR ligands that induced miR-155 also required myeloid differentiation factor 88 (MyD88) or the TIR-domain-containing adapter-inducing IFN-*β* (TRIF)-associated TLR3/TLR4 molecules. Furthermore, inhibition of c-Jun-N-terminal kinase (JNK) blocked miR-155 induction induced by [poly (I:C)] or TNF-*α*. 

 These initial studies provided the impetus for considering miRs as potential novel experimental targets in inflammatory arthritis. In that regard, Nakasa et al. [110] showed that mature miR146a and primary miR146a/b were highly expressed in RA synovial tissue where it was principally found in CD68^+^ macrophages with a smaller percentage of CD3^+^ T-cells and CD79a^+^ early progenitor B-cells also producing miR146a/b. In vitro miR146a/b was markedly upregulated in RA-SF by TNF-*α* and IL-1*β*, an indicator that high levels of these proinflammatory cytokines found in RA synovial fluid were likely to be responsible for the elevated level of miR146a/b found in RA synovial tissue. Stanczyk et al. [111] confirmed the basic findings of O'Connell et al. [108] and Nasaka et al. [110] and further showed higher levels of miR-146a and miR-155 in RA synovial tissue compared to synovial tissue from patients with osteoarthritis. Furthermore, Stanczyk et al. [111] showed that forced expression of miR-155 repressed the levels of MMP-3 (stromelysin-1) produced by RA-SF. miR-155 also reduced the expression of MMP-1 (collagenase-1) and MMP-3 induced by TLR ligands, TNF-*α*, IL-1*β*, [poly (I:C)] and bacterial lipoprotein. More recently, elevated expression of miR-146a, miR-155, miR-132, miR-16, but not miR-let-7a expression was found in RA synovium [112] with elevated miR-146a levels also found in circulating RA monocytes. Interestingly, TRAF6, and IRAK-1, two targets of miR-146a [107] were produced at similar levels in RA and control monocytes, despite the finding of increased miR-146 expression by RA monocytes. This result suggested that miR-146a produced by RA monocytes was apparently nonfunctional. As such, defective miR-146a could contribute to the dysregulation of TNF-*α*-signaling in RA since repression of TRAF6 and IRAK-1 in THP-1 cells resulted in an 86% reduction in TNF-*α*. Finally, Nagata et al. [85] showed in an autoantibody-induced model of murine inflammatory arthritis in DBA/1 mice that double-stranded miR-15a coadministered intra-articularly with FAM-atelocollagen complex resulted in elevated expression of miR-15a in the arthritic mouse synovium compared to mice injected with siRNA-atelocollagen complex representing the control group. Interestingly, the antiapoptotic protein bcl-2 was downregulated by miR-15a whereas caspase-3 was increased by miR15-a in the mice with autoantibody-induced arthritis compared to the control group. These results suggested that experimental therapy with miR-15a was capable of inducing caspase-3-dependent apoptosis in RA synovium by suppressing bcl-2 expression.

## 8. Tumor Necrosis Factor-Related Apoptosis-Inducing Ligand Receptors

 The failure of apoptosis to rectify aberrant cellular proliferation signals that activate synoviocytes, T- and B-cells, macrophages and osteoclasts is a signature event in various forms of inflammatory arthritis [36, 37]. Several intracellular processes have been identified as being defective in the regulation of apoptosis in inflammatory arthritis. These include, the prominence of the PI3K/PTEN/Akt cell survival pathway [113, 114], upregulation of HSP70 gene expression [115], suppressed Fas/CD95-mediated signaling [116], activation of NF-*κ*B [117, 118] by the proinflammatory cytokines, IL-1*β* and TNF-*α* [119], by reactive oxygen species and nitrogen reactive species [120] as well as the activity of antiapoptotic BH3-proteins of the bcl-2 family [37, 121], all of which dampen the apoptosis signaling cascade. Despite the potential that these cellular events could be targeted for intervention in inflammatory arthritis, recent attention has been mainly focused on the critical role of tumor necrosis factor- (TNF-) related apoptosis-inducing ligand (TRAIL) as a target for elevating the frequency of apoptosis in immune-mediated inflammatory cells primarily because agonist TRAIL-receptor-specific antibodies have shown encouraging results in promoting apoptosis in cancer cells in some clinical trials [122].

 Morel et al. [123] first showed that TRAIL induced RA-SF proliferation through activation of MAPK and PI3K/Akt since RA-SF proliferation could be inhibited by the ERK 1/2 SMI, PD98059, the p38 kinase SMI, SB203580 and the PI3K/Akt SMI, LY294002, respectively. TRAIL also induced apoptosis in about one-third of the cells but in the cells that survived, TRAIL promoted RA-SF proliferation. Of note, TRAIL-mediated RA-SF proliferation could be blocked by neutralizing anti-TRAIL antibody. Trichostatin A (TSA), a *Streptomyces *metabolite which specifically inhibits mammalian histone deacetylases was shown to sensitize RA-SF to TRAIL-induced apoptosis, whereas TRAIL or TSA alone were unable to induce RA-SF apoptosis [124]. The results of this study suggested that the initial small apoptotic response of RA-SF to TRAIL reported by Morel et al. [123] may have been due to the presence of inactive sites in the cascade of TRAIL-signaling that were uncovered by administering TSA which resulted in an increase in the frequency of apoptotic cells. 

 Monocyte production of IFN-*γ* also appeared to be critical in the dampening of TRAIL-mediated apoptosis in RA [125]. In that study which employed RA-FLS, IFN-*γ* rapidly phosphorylated STAT-1, -3 and -6 as well as ERK 1/2 but not Akt or NF-*κ*B p65. Interestingly, chemical inhibition of ERK 1/2 by PD98059 failed to overcome IFN-*γ*-induced suppression of TRAIL-mediated apoptosis. Furthermore, IFN-*γ*-mediated inhibition of TRAIL-signaling could not be accounted for by significant changes in TRAIL-R, procaspases-3,-8,-9, Fas-activated death domain protein (FADD), tumor necrosis factor receptor 1-associated death domain protein, silencer of death domain protein, FADD-like-interleukin-1*β* converting enzyme (FLICE) inhibitory protein, bcl-2, Bcl-xL or Bax activity. These results suggested that IFN-*γ* probably caused apoptosis resistance in RA-FLS in response to TRAIL by activating the JAK/STAT pathway. Although the precise mechanism accounting for IFN-*γ*-induced suppression of TRAIL-signaling beyond activation of STAT proteins in RA-FLS remains unknown, several more recent studies have addressed other aspects of apoptosis resistance in continuously proliferating cancer cells induced by INF-*γ*, Thus, Bonmort et al. [126] showed that DCs also synthesize INF-*γ*. Further, inhibitors of the c-kit tyrosine kinase in combination with IL-2 were shown to promote DC-mediated cytotoxicity of these tumor cells by TRAIL-mediated and IFN- type IIR-mediated pathways. 

 Epigenetic silencing of caspase-8 activity can also result in apoptosis resistance. In that regard, Häcker et al. [127] showed that inhibitors of histone deacetylase activity, including, valproic acid, suberoylanilide hydroxamic acid/Vorinostat, and MS-275 cooperate with IFN-*γ* to upregulate caspase-8 in medulloblastoma cells lacking caspase-8, and restore TRAIL-mediated cytotoxicity. In future studies, it will be interesting to determine the extent to which valproic acid, suberoylanilide hydroxamic acid or MS-275 can restore IFN-*γ* suppression of TRAIL-mediated signaling in RA-FLS. 

 In another line of investigation, Audo et al. [128] recently showed that TRAIL-induced apoptosis was inhibited in RA-FLS by the overexpression of the cell survival regulator proteins, p21, X-inhibitor of apotosis protein, MCP-1 and receptor-interacting protein. Furthermore, caspase-8 played a crucial role in mediating TRAIL-induced RA-FLS proliferation. Of note, TRAIL was shown to induce the breakdown of p21 and p27 that were both caspase-dependent, but independent of ERK 1/2, p38 kinase and PI3K/Akt activity. Additionally, Pundt et al. [129] showed that FasL- and TRAIL-mediated apoptosis in RA-SF cultures was cell-cycle dependent. Thus, future studies should take into consideration the pleiotypic nature of TRAIL-mediated signaling as well as perhaps targeting cell cycle-regulating genes in order to more appropriately define interventional strategies for employing TRAIL agonists in vivo. 

 Gene transfer and intra-articular administration of TRAIL have recently been employed to test the extent to which these strategies could modulate TRAIL-mediated apoptosis resistance in RA. Terzioglu et al. [130] employed adenoviral human TRAIL (i.e., Ad5hTRAIL) gene transfer in an attempt to overcome TRAIL-induced apoptosis resistance in primary synovial cell cultures obtained from RA patients. They reported that TRAIL-R was permissive for TRAIL-induced apoptosis. However, high levels of TRAIL-R4 decoy receptor expression correlated with TRAIL resistance. Of note, decoy receptor-2 (DcR2) a TRAIL-R and a target for p53 siRNA [131], but only in combination with Ad5hTRAIL, eliminated the TRAIL-induced resistance to apoptosis in these cells to a more significant extent than when the cells were transfected with Ad5hTRAIL alone. Thus, the results of this study suggested that modulation of TRAIL-R expression could enable RA synoviocytes to be sensitized to TRAIL-mediated signaling. Furthermore, TRAIL gene transfer may be a useful strategy to test in animal models of RA to examine the extent to which apoptosis induction in synoviocytes ameliorates arthritis severity. Employing a different experimental strategy, Yao et al. [86] attempted to induce synovial cell apoptosis in a rabbit model of experimental arthritis induced by intra-articular administration of allogeneic fibroblasts that were genetically engineered to oversecrete human IL-1*β*. Arthritic rabbits were then treated either by intra-articular administration of recombinant chimeric human TRAIL (rhTRAIL) or with saline. The TUNEL assay demonstrated extensive synovial cell apoptosis in the rabbits treated with rhTRAIL compared to the saline control group and a 50% reduction in lymphocyte infiltration in the inflamed joint. Furthermore, intra-articular administration of rhTRAIL altered neither the proteoglycan content of articular cartilage nor liver function. Taken together, the results of these studies suggested that a gene transfer strategy approach, keeping in mind the appropriate caveats needed to extend these experimental studies to clinical use [132], or administration of rhTRAIL might be capable of overcoming synoviocyte apoptosis resistance in RA.

## 9. Inhibition of Proteasome Activity

**Figure 5:**
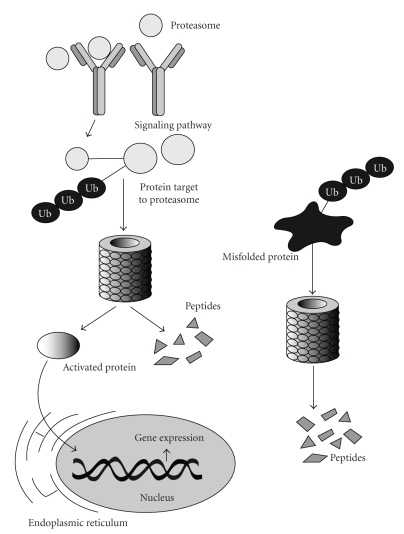
The Proteasome Signaling Pathway. Normally produced (left) or misfolded (right) proteins that are destined for 28S proteasome-mediated degradation are targeted for degradation either by phosphorylation (P) or after tagging with ubiquitin (Ub), via ubiquitin ligase [133]. In this pathway, a polyubiquitin protein chain becomes bound to the 28S proteasome providing a mechanism required for protein degradation. Nonphosphorylated or non-Ub-tagged proteins (i.e., “activated proteins”) can bypass the proteasome pathway and be transported to the nucleus.

 Proteasomes are large protein complexes that reside in both the nucleus and cytoplasm of eukaryotic cells [134]. A principal function of the 26S-proteasome is to regulate the concentration of completed proteins within a cell as well as to participate in the controlled degradation of misfolded proteins that is independent of enzyme activity within lysosomes [135] (Figure 5). In that regard, proteins destined for degradation in the proteasome are tagged with ubiquitin via a reaction that is catalyzed by ubiquitin ligases. A polyubiquitin chain is formed which becomes bound to the proteasome and provides the required structural component for initiating proteasome-mediated degradation. Because the proteasome regulates so many of the fundamental cellular processes that may result in aberrant proteins participating in cell cycle progression, proliferation, protein trafficking, apoptosis and immune-mediated inflammatory processes [135], inhibition of the proteasome pathway has become a promising focus of research for modulating key components of dysfunctional protein function in cancer and immune cell signaling [136–138]. 

 Prosurvival cell growth factors including, mutated p53, the E3 ubiquitin ligase hrd1, the ubiquitin-like protein sentrin, and NF-*κ*B were found to be increased in the pannus of RA synovial joints [139]. Thus, the increased concentration of these molecules is likely to be responsible for the fact that apoptosis is rarely detected in this tissue [36, 37]. Additionally, the continued uncontrolled growth of RA pannus despite the use of potent conventional and biologic DMARDs is likely to be responsible for the accelerated destruction of cartilage and bone in this disorder [38]. 

 PS-341 (bortezomib) is a newly developed proteasome inhibitor which induced apoptosis in cancer cells by inhibiting the activity of the Forkhead BoxM1 (FoxM1) transcription factor [140]. In that regard, overexpression of FoxM1 was shown to specifically override bortezomib-induced-apoptosis, but not doxorubicin-induced cancer cell apoptosis. More recently, bortezomib was shown to activate the mitochondrial pathway of apoptosis in activated CD4^+^ T-cells which was characterized by the accumulation of proapoptotic proteins, including, p53 upregulated modulator of apoptosis (PUMA), the bcl-2 protein family members, Noxa and Bim as well as p53 in the mitochondrial outer membrane [141]. Bortezomib also induced apoptosis and inhibited NF-*κ*B-dependent cytokine production by activated T-cells isolated from RA patients [142]. These cytokines included, TNF-*α*, IL-1*β* and IL-6 and IL-10. However, the effect of bortezomib on regulating the ratio of accumulated proinflammatory and antiinflammatory cytokines via inhibition of NF-*κ*B [143] in vitro will require further study. 

 In addition, the extent to which bortezomib might alter the activity of the newly described “secreted osteoclastogenic factor of activated T-cells” (SOFAT), a factor relevant in bone destruction in RA, distinct from RANKL [144], should be examined. 

 The use of putative proteasome inhibitors for regulating autoimmune arthritis-related pathophysiology was further complicated when Kwak et al. [145] recently showed that mg132, a selective proteasome inhibitor, (*K*_*i*_ = 4 nM) that inhibits I*κ*B degradation and N-Acetyl-Leu-Leu-Nle-CHO (ALLN), a calpain inhibitor, both enhanced ERK 1/2 and Akt activation resulting in osteoclast survival under conditions of survival factor deprivation. 

 In murine CIA, bortezomib administered by intraperitoneal injection significantly reduced the severity of arthritis and stabilized joint structure architecture while also suppressing the production of several biomarkers of immune-mediated inflammation, including, TNF-*α*, IL-1*β*, and IL-6, COX-2, and inducible nitric oxide synthase (iNOS) as well as inhibiting MMP-3 synthesis [87]. No blood cell, liver or kidney toxicity from was reported in the CIA mice treated with bortezomib. These results suggested that proteasome inhibition is worthy of additional study to determine the extent to which it may become a useful adjunctive strategy for regulating immune-mediated inflammatory arthritis.

## 10. Conclusions

 In vitro cultures of immune cells and synovial fibroblasts from nonarthritic and RA patients as well as well-validated animal models of inflammatory arthritis have been employed to test novel experimental strategies for attenuating proinflammatory cytokine production, MMP biosynthesis, T-cell and B-cell activity and inducing apoptosis as well assessing some compounds for their capacity to dampen the severity of clinical measures of arthritis. The discovery of promising specific targets for intervention in the inflammatory arthritis process have included, S1P, IL-7R, Syk, Raf/MEK/ERK 1/2, MK5/PRAK, miRNA, PI3K/PTEN/Akt, NF-*κ*B, TRAIL/TRAIL-R and the 28S proteasome. Altering the biological activity of several of these targets has shown efficacy for reducing inflammation-related pathways especially when employed in animal models of inflammatory arthritis (Table 1). The results of these studies make it highly likely that the discovery of some of these novel targets will eventually make their way into arthritis clinical trials. 

## Figures and Tables

**Table 1:** Some novel experimental therapies alter the severity of arthritis in animal models.

Target	Drug	Animal Model	Results	Reference
Spleen Tyrosine Kinase	R788/R406	Murine CIA^†^	↓ Arthritis	[68]
			↓ Cytokines	
MEK	PD184352	Murine CIA	↓ Arthritis	[82]
			↓ Proteoglycan degradation	
ERK	FR180294	Murine CIA	↓ T cell activation	[83]
miR-15a	miR-15a	Murine Autoantibody^‡^	↑ Apoptosis	[85]
			↓ bcl-2	
			↑ caspase-3	
TRAIL	rhTRAIL*	Rabbit IL-1*β*-transfected fibroblasts**	↑ Apoptosis	[86]
			↓ Lymphocyte infiltration	
Proteasome	Bortezomib	Murine CIA	↓ Arthritis	[87]
			↓ TNF-*α*,IL-1*β*, IL-6,iNOS, COX-2	
			↓ MMP-3	

^†^Type II Collagen-Induced Arthritis

^‡^FAM-atelocollagen + miR-15a

*Recombinant human TRAIL

**IL-1*β*-transfected human fibroblasts engrafted into rabbit synovial joint.

## Abbreviations

BCR: B-cell receptor
BH3: Bcl-2 homolog-3
COX-2: Cyclooxygenase-2
CREB: Cyclic AMP responsive element binding protein
CTLA-4: Cytotoxic T-lymphocyte antigen-4
DcR-2: decoy receptor-2
DMARD: Disease modifying antirheumatic drug
FADD: Fas-activated death domain
FLICE: FADD-like interleukin-1 converting enzyme
FoxM1: Forkhead box M1
FoxP3: Forkhead box P3
GPCR: G-protein coupled receptor
IL-1*β*: interleukin-1*β*
IL-6/IL-6R: Interleukin-6/Interleukin-6 receptor
IL-7/IL-7R: Inteleukin-7/Interleukin-7 receptor
INF-*β*/*γ*: interferon-*β*/*γ*
IRAP: interleukin-1 receptor antagonist protein
iNOS: inducible nitric oxide synthase
IRAK-1: IL-1 receptor-associated kinase-1
JAK/STAT: Janus kinase/signal transducers and activators of transcription
JIA: Juvenile idiopathic arthritis
JNK: C-Jun-amino terminal kinase
KC-Gro-*α*: Keratinocyte cotton rat growth-regulated protein-*α*
MCP-1: macrophage chemoattractant protein-1
miRNA: microRNA
MK5/PRAK: mitogen-activated protein kinase 5/p38 kinase regulated/activated protein kinase
MMP: matrix metalloproteinase
MyD88: myeloid differentiation factor-88
NF-*κ*B: nuclear factor-*κ*B
PI3K/PTEN/Akt: Phosphoinositide-3-kinase/phosphatase and tensin homolog/serine-threonine kinase Akt
PGE_2_: Prostaglandin E_2_
RA: Rheumatoid arthritis
RA-FLS: Rheumatoid arthritis-fibroblast-like synoviocytes
Raf/MEK 1/2/ ERK 1/2: Raf/MAPK kinase 1/2 /extracellular signal-regulated kinase 1/2
RANKL: receptor activator of nuclear factor *κ* *B* ligand
RISC: RNA-induced silencing complex
SAP/MAPK: Stress-activated protein/mitogen-activated protein kinase
SMI: small molecule inhibitor
SOFAT: Secreted osteoclastogenic factor secreted of activated T-cells
SphK-1/2: Sphingosine kinase-1/2
S1P: sphingosine-1-phosphate
SP1L: Sp1 lyase
SyK: spleen tyrosine kinase
TCR: T-cell receptor
TLR: Toll-like receptor
TNF-*α*: Tumor necrosis factor-*α*
TRAF-6: Tumor necrosis factor-associated factor-6
TRAIL: Tumor necrosis factor-related apoptosis-inducing ligand
ZAP-70: *ζ*-chain-associated protein 70.
